# An aerobic exercise intervention to improve metabolic health among people living with HIV with at-risk alcohol use: the ALIVE-Ex research study protocol

**DOI:** 10.1186/s12981-023-00530-2

**Published:** 2023-06-09

**Authors:** Liz Simon, Stefany D. Primeaux, Danielle E. Levitt, Brianna Bourgeois, Neil M. Johannsen, Adrianna Peters, Jameel Ahmed, Richard H. Marshall, Alexandra H. Fairchild, Tekeda F. Ferguson, Patricia E. Molina

**Affiliations:** 1 Department of Physiology, LSU Health Sciences Center, 1901 Perdido Street, MEB/7205, New Orleans, LA 70112 USA; 2 Comprehensive Alcohol HIV/AIDS Research Center, LSU Health Sciences Center, New Orleans, LA 70112 USA; 3 Joint Diabetes, Endocrinology & Metabolism Program, Pennington Biomedical Research Center, Baton Rouge, LA 70808 USA; 4 School of Kinesiology, Louisiana State University, Baton Rouge, LA 70803 USA; 5 Department of Medicine, LSU Health Sciences Center, New Orleans, LA 70112 USA; 6 Department of Radiology, Tulane University School of Medicine, New Orleans, LA 70112 USA; 7 Department of Radiology, LSU Health Sciences Center, New Orleans, LA 70112 USA; 8 Department of Epidemiology, LSU Health Sciences Center, New Orleans, LA 70112 USA; 9 Present Address: Department of Kinesiology & Sport Management, Texas Tech University, Lubbock, TX 79409 USA

**Keywords:** People living with HIV, Alcohol, Aerobic exercise protocol, Dysglycemia

## Abstract

**Background:**

Effective antiretroviral therapy (ART) in people living with HIV (PLWH) has improved life expectancy and increased risk of age-associated cardiometabolic comorbidities. At-risk alcohol use is more frequent among PLWH and increases the risk of health challenges. PLWH with at-risk alcohol use are more likely to meet criteria for prediabetes/diabetes and this is associated with impaired whole-body glucose-insulin dynamics.

**Methods:**

The Alcohol & Metabolic Comorbidities in PLWH: Evidence Driven Interventions Study (ALIVE-Ex Study, NCT03299205) is a longitudinal, prospective, interventional study to determine the effects of an aerobic exercise protocol on improving dysglycemia among PLWH with at-risk alcohol use. The intervention is a moderate intensity aerobic exercise protocol implemented 3 days per week for 10 weeks at the Louisiana State University Health Sciences Center-New Orleans. Participants who have a fasting blood glucose level between 94 and 125 mg/dl will be enrolled in the study. Oral glucose tolerance tests, fitness assessments, and skeletal muscle biopsies will be performed pre- and post-exercise intervention. The primary outcome is to determine whether the exercise protocol improves measures of whole-body glucose-insulin dynamics, cardiorespiratory fitness, and skeletal muscle metabolic and bioenergetic function. Secondary outcomes are to determine whether the exercise intervention improves cognitive function and overall quality of life. Results generated will demonstrate the effect of exercise on glycemic measures in PLWH with subclinical dysglycemia and at-risk alcohol use.

**Conclusions:**

The proposed intervention will also have the potential to be scalable to promote lifestyle changes among PLWH, particularly in underserved communities.

## Background

With effective antiretroviral therapy (ART), people living with HIV (PLWH) have near normal life expectancy and increased risk of age-associated comorbidities. Cardiometabolic comorbidities [1–6], are among the leading causes of morbidity and mortality among PLWH [1–5, 7, 8]. At-risk alcohol use is more frequent among PLWH than the general population and increases the risk of health challenges [9–11]. Our multidisciplinary research team is conducting a longitudinal clinical observational study, the New Orleans Alcohol and HIV study (NOAH) [12], to examine the impact of at-risk alcohol use on risk for comorbidities in PLWH. The study population is comprised of majority socioeconomically disadvantaged and majority Black/African American individuals in the Greater New Orleans Area. Data indicate that 57% have at-risk alcohol use [12], ~ 36% meet criteria for metabolic syndrome, ~ 50% have insulin resistance as measured by the Homeostatic Model Assessment of Insulin Resistance (HOMA-IR), and ~ 15% meet criteria for type 2 diabetes[13]. Results also showed a significant negative association between HOMA-β, a surrogate indicator of pancreatic cell function, and multiple measures of alcohol use including Alcohol Use Disorders Identification Test (AUDIT)-C, 30-d Timeline Followback (TLFB), and a biomarker of recent alcohol use Phosphatidylethanol (PEth) [13].

Previously, studies in simian immunodeficiency virus (SIV)-infected macaques, a preclinical model of HIV, showed that chronic binge alcohol (CBA) significantly impaired endocrine pancreatic response to a glucose load [14], and increased pathophysiological alterations in multiple metabolically active organs [15]. CBA administration also increased skeletal muscle proteasomal activity, impaired anabolic pathways and insulin signaling [16–19] and altered mitochondrial bioenergetic function [20, 21]. Together, these findings suggest that at-risk alcohol use increases risk of metabolic dysregulation in PLWH, and in a preclinical model of HIV infection, highlighting the importance of developing interventions to ameliorate disease burden. Specifically, strategies to reduce cardiometabolic comorbidities and improve overall quality of life that can be scalable and can be implemented in clinical and community settings are urgently required to mitigate health disparities.

Evidence suggests that people engaging in regular aerobic exercise preserve or improve glycemic control, cardiovascular function, skeletal muscle function, have reduced risk of metabolic syndrome, and improved quality of life [22, 23]. Specifically, structured walking for approximately 30 min three times per week reduces fasting glucose, hemoglobin A1c, and HOMA-IR [24, 25]. Moreover, PLWH engaging in exercise have increased energy, improved self-concept, increased physical fitness, mental health, improved cardiometabolic measures, body composition and immune function [23, 26–30]. Studies show that moderate intensity exercise (40–60% heart rate reserve [HRR]) improves mitochondrial dysfunction, insulin resistance and diabetes among PLWH [31]. Additionally, our studies in the preclinical SIV model provided evidence that ex vivo β2 adrenergic stimulation of muscle myoblasts improved mitochondrial bioenergetics and mitochondrial copy number [21] supporting the hypothesis that aerobic exercise may improve mitochondrial homeostatic mechanisms resulting in improved glycemic control. Despite compelling evidence that exercise can be an effective strategy to improve metabolic health, little is known of how exercise improves metabolic health in PLWH, particularly those with at-risk alcohol use.

Based on our preclinical and clinical observational studies and evidence from the literature, we launched the Alcohol & Metabolic Comorbidities in PLWH: Evidence Driven Interventions Study (ALIVE-Ex Study, NCT03299205). This is an ongoing longitudinal prospective, interventional study investigating the effects of aerobic exercise in PLWH with fasting dysglycemia and at-risk alcohol use. The study consists of two phases. The objective of the first phase was to determine whether PLWH with at-risk alcohol use had increased risk of dysglycemia and dysregulated muscle metabolic function. The overall objective of the second phase of the study is to determine whether moderate intensity aerobic exercise will improve measures of whole-body glucose insulin dynamics and improve skeletal muscle metabolic and bioenergetic function. The secondary objective of Phase 2 is to determine whether the exercise intervention improves cognitive function and overall quality of life.

## Methods

### Study overview

The ALIVE-Ex study is an aerobic exercise intervention (NCT03299205) conducted among PLWH in the Greater New Orleans Area. The study was approved by the Institutional Review Board of Louisiana State University Health Sciences Center-New Orleans (LSUHSC-NO # 736) was launched in 2017 and is expected to continue through 2023.

### Study setting

Initially, ALIVE-Ex leveraged participants from the NOAH study. NOAH participants are a clinic-based sample of PLWH aged ≥ 18 years under care at the HIV outpatient clinic of the University Medical Center-New Orleans (UMC-NO) [12]. In 2018, the ALIVE-Ex study expanded recruitment beyond NOAH to the community of the Greater New Orleans area.

### Recruitment

Potential participants were invited to voluntarily join the study. Study enrollment started in November 2017 and is anticipated to stop in May 2023. Based on the eligibility criteria, letters were sent to NOAH participants inviting them to the study. Other recruitment efforts included posting study flyers in local pharmacies, HIV clinics, local HIV Community Advisory Boards, and relevant community events. Past and ongoing recruitment efforts also include invitations during clinic visits, advertisements, use of social media, health care provider referrals, and presenting the study at local HIV community advisory boards.

### Consent

Study purpose, details, risks, and benefits are thoroughly explained to participants and any questions answered. Participants must verbally demonstrate understanding and provide written informed consent before inclusion in the study. Participants receive compensation for all testing procedures and exercise sessions.

### Study timeline

**Fig. 1:**
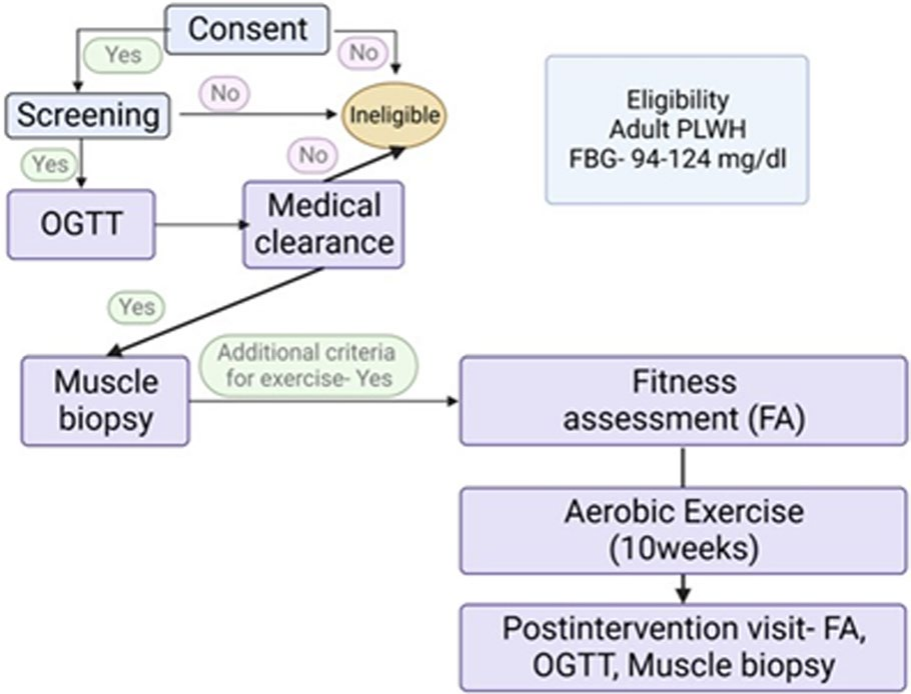
Study timeline for the Alive-Ex research protocol

The study timeline is as shown in Fig. 1. The eligibility criteria are shown in Table 1.

**Table 1:** Inclusion and exclusion criteria for the ALIVE-Ex Study

Inclusion criteria	Exclusion criteria
PLWH ≥ 18 years of age	Diagnosed diabetes (self-reported)
Fasting plasma glucose between 94 and 124 mg/dl	Lack of informed consent
Answered “No” to all questions of PAR-Q, with the exception that if participants answered “yes” to taking medications for high blood pressure, they were evaluated at medical clearance and enrolled into the study, based on the discretion of the cardiologist	Non-English speaking
	Females who are pregnant
	Acute alcohol intoxication on any visit days as determined by breathalyzer
	Additional exclusion criteria for exercise intervention
	Orthopedic or cardiovascular complications that preclude from exercise
	Peripheral neuropathies
	Allergy to lidocaine
	Use of anticoagulant medication (blood thinners)
	Acute illness within the preceding six weeks of study visit (fever, new antibiotic use, or unscheduled healthcare visit (for illness)

### Screening

After informed consent, participants are instructed to fast and not drink alcohol overnight. Participants are considered eligible if they have a fasting blood glucose of 95–124 mg/dL, measured by a single-use auto-disabling finger stick device and glucose meter (True Metrix® Pro Blood Glucose Monitoring System, McKesson).

### Oral glucose tolerance test (OGTT)

Blood is collected at time 0, after which participants ingest a calibrated dose (10 oz) of a glucose solution (Trutol; 75 g glucose) over a 5-to-15-min period. Blood is collected at 1 and 2 h following the glucose drink. During that time, participants are seated without eating, drinking, or smoking and complete the following questionnaires: demographics, AUDIT, and TLFB. At the end of the OGTT, participants are monitored for signs of hypoglycemia. OGTTs are performed at two timepoints, the first one at least a week before the first exercise session and the second 3 days after the last exercise session.

### Medical clearance

A cardiologist affiliated to LSUHSC-NO provides medical clearance for eligibility for exercise intervention. Approval for subject participation in the exercise protocol is based on absence of significant cardiovascular impairments (i.e., angina, clinically significant arrhythmias, uncontrolled hypertension); absence of significant peripheral neuropathies or conditions that would increase risk of falls or limit ability to safely exercise on a treadmill; and absence of active cocaine or amphetamine use. The resting heart rate (RHR) recorded during the electrocardiogram (ECG) is retained and used for HRR calculation.

### Muscle biopsy

Participants are instructed to fast for 9 h prior to the biopsy visit. TLFB, blood pressure, weight, and waist and hip circumference are collected. Following the 0 h blood collection, participants consume a defined (calorie and nutrient composition) meal (i.e., Ensure, Carnation Instant Breakfast). Ninety (90) minutes later, a skeletal muscle biopsy is performed under ultrasound guidance by interventional radiologists associated with LSUHSC-NO. Using sterile technique, 2 cc of 2% lidocaine is injected at the incision site on the right vastus lateralis muscle. A 14-gauge core needle is inserted through a single-entry point. Samples of the vastus lateralis are obtained on 6 passes. Pressure is held on the biopsy site for 5 min, followed by pressure dressing for 1 h. After that time, the dressing is removed. Participants are contacted 24 h later to inquire about any adverse events and reminded to contact study personnel if any adverse events were to occur. Muscle biopsies are performed at two timepoints, the first one at least a week before the first exercise session and the second 5 days after the last exercise session.

### Fitness assessment

Approximately 5–7 days following the pre-intervention muscle biopsy, cardiorespiratory fitness (CRF) is assessed on a treadmill at the LSUHSC-NO Wellness Center using a modified Balke protocol. Following a brief warm-up period, participants begin the assessment by walking at brisk pace and level grade for 2 min, after which grade is increased 2% every 2 min until the computer software recommends stopping the assessment or volitional exhaustion is achieved. Estimated maximal oxygen uptake (VO_2max_) is calculated from the exercise test. In addition to VO_2max_, estimated metabolic equivalents (METs) are calculated from the speed and maximal grade achieved during the exercise test using standard equations from the American College of Sports Medicine’s Guidelines for Exercise Testing and Prescription (ACSM’s Guidelines for Exercise Testing and Prescription, 9th ed. 2014). Heart rate is monitored throughout the test to ensure safety and for accurate prescription of the exercise intervention. After the conclusion of the test, the participant goes through a cool down period. A wall push-up test is also performed to assess upper body muscular endurance. A personalized treadmill exercise prescription is developed based on the RHR recorded during the ECG. The heart rate responses to the graded exercise test for each participant is determined to achieve a level of moderate intensity (40–60% HRR) during the exercise program. Target heart rate (THR) ranges are calculated using the following equation: THR = %HRR * (HR_max_—RHR) + RHR, where %HRR is the percentage of HRR that defines the lower and upper ends of the target range (i.e., 0.40, 0.50, or 0.60). HR_max_ is the age-predicted maximum heart rate (220—age [yrs]). Using this data, an initial speed and incline for treadmill exercise is prescribed for each participant and adjusted as needed to achieve the desired exercise intensity for each exercise session. While speed and incline both increase throughout the intervention period, participant preferences dictate which variable increases first and more frequently. The participants are also provided with an accelerometer (i.e., Fitbit Zip) to monitor daily activity and steps. The fitness assessments are performed at two timepoints and constitute the first exercise session and the last exercise session.

### Exercise intervention

The moderate intensity aerobic exercise protocol is implemented 3 days per week for 10 weeks at the LSUHSC-NO Wellness Center. Each exercise session lasts for 30–42 min on a treadmill. The first and last 6 min are dedicated to warm-up and cool-down, respectively. The exercise begins at the lower end of the moderate intensity range (40–50% of HRR) for the first 4 weeks with progressive increases in duration. Beginning week 5, participants progress to the higher end of the moderate intensity range (50–60% of HRR). The duration of exercise sessions remains constant after week 4 so the absolute dose of exercise is altered through increasing intensity, i.e., from low-moderate to more vigorous, by increasing grade and/or speed. The speed, grade, heart rate and rating of perceived exertion (RPE) is monitored every three minutes during every session. The 24 h food recall questionnaire (ASA24), quality of life questionnaire (SF-36), Hospital depression and anxiety questionnaire, and Montreal Cognitive Assessment Test (MoCA) are administered before and after the 10-week exercise intervention period. The exercise sessions are one-on-one, and the participants appreciate the social interactions with the personnel who perform the sessions. Participants are contacted via their preferred method of communication (text or phone call) to remind them of each appointment. They are contacted if they do not come for the appointment to ascertain the reason and reschedule the visit. Participants are required to complete at least 80% of the sessions within the 10-week exercise intervention period. Participants are allowed to reschedule sessions within the same week. However, if participants miss 6 consecutive exercise sessions without rescheduling, they are removed from the study. Study personnel also send motivational texts to the participants. The incentive for exercise sessions was increased to account for the increased costs associated with transportation and to cover additional incidental expenses.

### Criteria for modifying exercise protocol

Inability of participants to use the treadmill, such as orthopedic limitations, is addressed by conducting the sessions using a stationary bike with moving arms, and the exercise intensity is matched to that of using the treadmill.

### Outcome measures

#### OGTT

Outcome measures are determined as previously described [32]. Briefly, plasma glucose is measured at 0, 1 and 2 h using a glucose Analox analyzer (GM7 Analox microstat, Analox Instruments, USA) and area under the curve calculated. Plasma insulin and C-peptide are measured at 0, 1 and 2 h using ELISAs (Millipore Human Insulin ELISA, cat.no. EZHI-14 K; Alpco C-Peptide ELISA, cat.no. 80-CPTHU-E10.1). HOMA-IR is calculated (fasting glucose (mg/dL) x fasting insulin (µU/mL) divided by 405) and HOMA-β is calculated as 60 × fasting insulin (μU/mL) / (fasting glucose (mg/dL)—63). High molecular weight adiponectin is measured in the baseline samples using an ELISA (Alpco HMW Adiponectin ELISA, cat.no. 80-ADPHU-E01).

#### Skeletal muscle biopsy

A portion of the muscle sample (~ 75 mg) is cleaned of residual blood, fat, and connective tissue and subsequently flash frozen. About 50 mg is used for myoblast isolation (described below), and approximately ~ 25 mg is immediately fixed in zinc-buffered formalin. Primary myoblasts are isolated from the muscle biopsies as previously described [33]. Briefly, muscle tissue is minced, and trypsin digested (0.25% trypsin EDTA diluted 1:4 in Ham’s F-12). Digested muscle tissue is plated in growth media [Ham’s F-12 nutrient mixture with 10% fetal bovine serum, 2% l-glutamine, and 2.5 ng/ml recombinant human fibroblast growth factor (R&D systems, Minneapolis, MN)] to allow fibroblasts to adhere to the plate. Supernatant containing muscle tissue and myoblasts is transferred to a fresh plate and myoblast colonies are allowed to grow for 1 week. Thereafter, media is changed every other day and passaged at 80–90% confluence and cryopreserved after each passage. Experiments are performed with myoblasts at passage (P)3 or P4.

#### Mitochondrial function

Mitochondrial oxygen consumption rate (OCR) is measured using a Mito Stress Test and Seahorse XFe96 technology (Agilent Technologies, Santa Clara, CA) as previously published [33]. Briefly, myoblasts are seeded in triplicate on a collagen-coated 96-well Seahorse plate (35,000 cells/well) and maintained under standard cell culture conditions. After 24 h, growth media is replaced with XF Assay Medium (pH 7.4) with sodium pyruvate (1 mM), l-glutamine (2 mM), and glucose (10 mM) and incubated at 37 °C without CO_2_ for 1 h before measuring myoblast OCR. Respiratory parameters are assessed by the sequential addition of oligomycin (1.5 µM), carbonyl cyanide‐p‐trifluoromethoxyphenylhydrazone (FCCP; 2 μM), and rotenone/antimycin A (0.5 μM). Resulting OCR measurements are normalized to cell count obtained by staining nuclei with Hoechst dye (2 µM; ThermoFisher Scientific, Waltham, MA) and visualizing on a BioTek Cytation 1 cell imaging multi-mode reader (BioTek, Winooski, VT).

#### Glycolytic function

To quantify glycolytic function, capacity, and reserve, a Glycolysis Stress Test is performed using Seahorse XFe96 technology. Myoblasts are plated in Seahorse plates with glucose- and pyruvate-free XF Assay Medium (pH 7.4) with L-glutamine (2 mM). Myoblasts are incubated at 37 °C without CO_2_ for 1 h before measuring myoblast extracellular acidification rate (ECAR) at baseline and after the sequential addition of glucose (10 mM), oligomycin (1.5 uM), and 2-deoxyglucose (2-DG; 50 mM). Measures are normalized to cell count as described above.

#### Mitochondrial volume

Myoblasts are incubated with growth media containing MitoTracker Deep Red FM (Invitrogen, Carlsbad, CA) (100 nM) under standard cell culture conditions for 30 min as previously published [33]. Cells are prepared for flow cytometry using the PerFix-nc Kit (Beckman Coulter, Brea, CA). Ten-thousand events per sample are acquired and excited by a 633 nm wavelength laser line using flow cytometry (BD FACSCanto II, Becton, Dickinson and Company, Franklin Lakes, NJ). Fluorescence is analyzed using FlowJo software (version 10.7.1, Becton, Dickinson, and Company) and the mean fluorescence intensity (MFI) of each experimental sample determined.

#### RNA and DNA isolation and quantitative real-time polymerase chain reaction (qPCR)

To assess expression of genes, and microRNAs, total RNA is extracted from flash frozen SKM samples (~ 10 mg) using the miRNeasy Mini Kit (Qiagen, Valencia, CA) and cDNA synthesized using the QuantiTect reverse transcription kit (Qiagen) [33]. Custom primers designed to span exon-exon junctions are purchased from Integrated DNA Technologies (Coralville, IA). qPCR reactions are carried out in duplicate using a CFX96 thermal cycler (Bio-Rad, Hercules, CA) with ribosomal protein S13 (*RPS13*) as the endogenous control. Data is analyzed using the 2^−ΔΔCt^ method.

#### Plasma microRNA expression

To assess the expression of circulating microRNAs, total RNA is extracted from 200 μL of fasting plasma using the miRNeasy serum/plasma kit (Qiagen) as previously described [34]. For normalization, 25 fmol of synthetic miR-cel-238 is added to each RNA sample. cDNA is synthesized using the Reverse transcription kit (Thermofisher) and mature miRs measured using TaqMan primers and Universal PCR Master Mix II (ThermoFisher, Waltham, MA). Serial dilutions of known concentrations of miRs (Integrated DNA Technologies, Coralville, Iowa) of interest are used to generate standard curves and miR expression represented as copy number per μL and normalized to copies of miR-cel-238.

#### Secondary outcomes

Changes in alcohol use are assessed using TLFB and Peth pre- and post-exercise intervention. Healthy Eating index (HEI) using Automated Self-Administered 24-h Dietary Assessment Tool (ASA24); Montreal Cognitive Assessment (MoCA); Hospital Anxiety and Depression Scale (HADS); and 36-Item Short Form Health Survey (SF-36) scores; pre and post exercise intervention are used to assess changes in dietary patterns, cognitive ability, and overall quality of life. The ASA24 is based upon the United States Department of Agriculture's (USDA) Automated Multiple-Pass Method (AMPM). Data from multiple validation and evaluation studies indicate good to strong agreement between the ASA24 system and standardized interviewer-administered 24-h recalls. Furthermore, data are well estimated for HEI scores based on the 24-h dietary recall [35]. The reliability of MoCA is 0.89, and the intraclass correlation coefficient of 0.955 and is shown to be good. Additionally, with a cut-off of 26 points, a sensitivity of 80% and specificity of 75% is reported [36]. The HADS has been found to perform well in assessing the symptom severity and presence of anxiety disorders and depression symptoms in both somatic, psychiatric, and primary care patients and in the general population [37]. Finally, the SF-36 was developed as a set of generic, coherent, and easily administered quality-of-life (QOL) measures. These measures rely upon participant self-reporting, provide a direct quantitative indication of an individual's health status, and is widely used as a QOL evaluation tool [38, 39].

### Data management and analysis

All data are entered into REDCap and are handled with confidentiality. Personal information of participants such as phone number and email addresses are used only to contact the participant. Names of participants appear only in the demographic form. The participants’ demographic and outcome measures of interest pre- and post-exercise intervention will be summarized using descriptive statistics. Mean, standard deviation, and range will be calculated for continuous variables, and frequencies and percentages will be generated for categorical variables. For continuous variables (such as VO_2max_, OCR, ECAR), the normal distribution assumptions will be tested. If necessary, the data will be transformed to follow the normal distribution assumption. If the normality assumption cannot be met, these variables will be classified as categorical or analyzed using non-parametric tests. For categorical variables (e.g., glucose at 2 h, HOMA-IR, HOMA-b) clinical cutoff values will also be used. The changes in the outcome measures (e.g., OGTT outcomes, bioenergetic measures, microRNA expression, RHR, VO_2max_, MOCA measures, ASA24 measures) between pre and post exercise intervention will be tested using the paired t-test. Linear regression models will also be used to analyze the relationship between alcohol use with the outcome measure changes as the dependent variable, adjusting for potential confounding factors (such as age, sex, race, viral load and CD4 counts). If there is matching or balance achieved, age and gender will be used as categorical variables rather than confounders. A p < 0.05 will be used for statistical significance.

#### Handling of missing data

To ensure that there is minimal missing data, several measures are in place such as appointment reminders, interviewer administered questionnaires instead of self-administered questionnaires, automated data entry systems that will not progress until data fields are completed, and robust standard operating procedures for biosample processing and analysis. In the instance of missing exercise sessions, outcomes will be analyzed using dose response and will consider the potential differences in outcome measures by level of exposure to the intervention. In addition, depending on the type of missing data for both primary and secondary outcome measures, a variety of methods such as imputation, omission, or analysis, will be used to limit the impact and reduce generalizability of data [40].

## Results

The first phase of the study that determined whether PLWH with at-risk alcohol use had increased risk of dysglycemia and dysregulated muscle metabolic function are published.

- Results indicate that in response to an oral glucose tolerance test (OGTT) in PLWH with fasting dysglycemia; participants with at-risk alcohol use were five times more likely to meet criteria for prediabetes/diabetes. At-risk alcohol use was negatively associated with fasting C-peptide levels, increased odds of decreased HOMA-β, and increased 2-h glucose values [32]. These changes in whole body glucose insulin dynamics were also associated with skeletal muscle mitochondrial bioenergetic dysfunction.
- Bioenergetic health index (BHI), a positive indicator of mitochondrial bioenergetic health, was lower, and non-mitochondrial oxygen consumption was higher with increasing AUDIT scores. Proton leak, indicating decreased ATP synthesis, was significantly associated with higher 2-h glucose levels and AUDIT score, and myoblast mitochondrial volume was positively associated with the interaction between 2 h glucose and AUDIT. In addition, expression of genes associated with lipid handling were significantly decreased with the interaction of 2-h glucose and AUDIT, and expression of genes controlling mitochondrial biogenesis and dynamics were significantly decreased with higher 2-h glucose levels and AUDIT scores, respectively [33]. All these findings indicate that skeletal muscle mitochondrial health and bioenergetic function are dysregulated with at-risk alcohol use and dysglycemia.
- We were also able to demonstrate that recent alcohol use (PEth) influenced associations between circulating microRNAs implicated in metabolic function. Expression of miR-206 (muscle enriched) was significantly lower, and expression of miR-let-7b and miR-146a (adipose enriched) were significantly higher in PLWH with positive PEth. Participants with no recent alcohol use had miR-133a (muscle enriched) and miR-221 (adipose enriched) expression levels associated with altered glucose/insulin dynamics. While in participants with positive PEth, miR-20a (liver enriched) and miR-375 (pancreas enriched) together strongly predicted increased 2 h glucose levels [34]. Thus, results indicate that associations between circulating miRs and measures of glucose/insulin dynamics are modulated by alcohol use suggesting unique pathophysiological mechanisms contributing to altered glucose homeostasis in PLWH.

At the time of submission of the manuscript, the second phase of the study is ongoing. Based on prescreening, or preliminary eligibility from the parent NOAH study, 395 PLWH were assigned an ALIVE-Ex specific ID, out of whom 318 came for consent visits. Of the 242 consented, 155 met eligibility criteria. The general demographics of the participants enrolled are shown in Table 2. One hundred and fifty-two (152) participants underwent pre-exercise OGTT and of these, 84 subjects underwent medical clearance. Sixteen subjects did not fulfill eligibility criteria as shown in Table 3. Sixty-three pre-muscle biopsies were performed and as of November 2022, 33 participants have completed exercise, post intervention fitness assessments, OGTTs and muscle biopsies.

**Table 2:** Demographic Parameters of the ALIVE-Ex Study

Demographics	(n = 155)
	% (n)
Sex	
Female	31.6(49)
Male	69 (107)
Race	
African American	76.8 (119)
White	20.6 (32)
Other	1.9 (3)
Age, years	
20 to 30	6.4 (10)
31 to 40	14.2 (22)
41 to 50	18 (28)
51 to 60	45.8 (71)
60 +	15.4 (24)
Income	
< $20,000	84.5 (131)
$20,000 to $39,999	9 (14)
$40,000 +	6.4 (10)
Education	
< High School	34.8 (54)
High School Graduate	28.3 (44)
Some College, Junior/Community College, Vocational/Trade School	26.4 (41)
4-year College/Graduate/Professional School	10.3 (16)

**Table 3:** Reasons for being ineligible for exercise of the ALIVE-Ex Study

Reason for ineligibility	Number
Active cocaine or amphetamine use	9
Angina	1
Lidocaine allergy	1
Uncontrolled hypertension	3
Orthopedic complications	2
Total	16

We anticipate that the exercise intervention will result in an increase in cardiorespiratory fitness reflected as decreased Resting Heart Rate (RHR) and increased VO_2max_. We also anticipate that the exercise intervention will improve OGTT outcome measures. We predict that the exercise will positively impact muscle bioenergetic measures and this will associate with overall changes in glycemic measures. We anticipate that exercise will have the greatest impact on these outcome measures in people with at-risk alcohol use. Although the intervention’s objective was not to improve other lifestyle choices such as diet and alcohol use, the analysis will identify whether exercise changes dietary patterns and alcohol use. It will also identify whether there is an improvement in cognitive measures and overall quality of life.

## Discussion and conclusions

PLWH have a high prevalence of physical inactivity, increased risk of cardiometabolic comorbidities [1–5], and at-risk alcohol use [41]. This is compounded by significant health disparities associated with race, ethnicity, socioeconomic status, and geographic location [42, 43]. Exercise is an effective intervention to improve cardiometabolic health across all populations [23, 44–46]. Studies also suggest that people with substance use disorders, when provided with structured exercise interventions derive general health benefits [47–51]. Results from our preclinical [14, 15, 52, 53] and clinical studies and from the first phase of this intervention study [32–34] indicate that PLWH with at-risk alcohol use have metabolic dysregulation.

The ALIVE-Ex study will be the first study that will determine the impact of a moderate intensity aerobic exercise intervention on metabolic health among PLWH with at-risk alcohol use. Not only will the participants derive direct benefits from the exercise regimen, but the results generated will provide knowledge on the effect of exercise on glycemic measures and muscle bioenergetic adaptations in people with subclinical dysglycemia. The proposed intervention will also have the potential to be scalable and our hope is that it may be recommended to promote lifestyle changes among PLWH particularly in underserved communities.

### Limitations and potential modifications considered for future interventions

Based on qualitative and pilot data analysis, we propose that incorporating resistance exercise into the ALIVE-Ex intervention will show enhanced benefits, improving measures of glucose homeostasis, mitochondrial health, and cardiometabolic measures among PLWH [54–58]. Our studies in the NOAH cohort indicate that decreased lean body mass is linked to frailty risk in PLWH [59] and we believe that incorporating resistance exercise may be particularly beneficial in preserving lean muscle mass, a metabolically active tissue [60, 61]. The current study does not perform strict dietary monitoring but will assess changes in dietary patterns. We also intend to perform secondary analysis based on HEI on impact of the exercise intervention on glycemic measures according to dietary habits of participants. Based on the results, future trials may propose implementing optimal dietary practices or interventions in this population. Compelling evidence suggests that decreasing alcohol use or increasing physical activity will be beneficial for improving and sustaining cardiometabolic health. Thus, we predict that combining a behavioral intervention with the structured exercise intervention will synergistically sustain physical activity and reduce alcohol use. This will have a profound impact on improving overall health particularly among the underserved communities. Finally, focus groups conducted will help refine setting, frequency, and additional factors that may enhance acceptance and feasibility of the intervention as we attempt to expand its implementation.

## Data Availability

The datasets generated and/or analyzed during the current study are not publicly available because it is an ongoing study but are available from the corresponding author on reasonable request.

## Abbreviations

ART: Antiretroviral therapy
PLWH: People living with HIV
NOAH: New Orleans alcohol and HIV study
HOMA-IR: Homeostatic model assessment of insulin resistance
AUDIT: Alcohol use disorders identification test,
TLFB: Timeline follow back
SIV: Simian immunodeficiency virus
CBA: Chronic binge alcohol
HRR: Heart rate reserve
ALIVE-Ex Study: Alcohol & metabolic comorbidities in PLWH: Evidence driven interventions study
LSUHSC-NO: Louisiana state university health sciences center-New Orleans
UMC-NO: University medical center-New Orleans
OGTT: Oral glucose tolerance test
ECG: Electrocardiogram
CRF: Cardiorespiratory fitness
VO_2max_: Maximal oxygen uptake
THR: Target heart rate
RPE: Rating of perceived exertion
MoCA: Montreal cognitive assessment test
OCR: Oxygen consumption rate
FCCP: Carbonyl cyanide‐p‐trifluoromethoxyphenylhydrazone
ECAR: Extracellular acidification rate
BHI: Bioenergetic health index
Peth: Phosphatidyl ethanol
HEI: Healthy eating index
